# The New Challenge of Green Cosmetics: Natural Food Ingredients for Cosmetic Formulations

**DOI:** 10.3390/molecules26133921

**Published:** 2021-06-26

**Authors:** Irene Dini, Sonia Laneri

**Affiliations:** Departemt of Pharmacy, University of Naples Federico II, Via Domenico Montesano 49, 80131 Napoli, Italy; slaneri@unina.it

**Keywords:** phytochemical analyses, food analyses, spices, condiments, seasonings, nutricosmetic

## Abstract

Nowadays, much attention is paid to issues such as ecology and sustainability. Many consumers choose “green cosmetics”, which are environmentally friendly creams, makeup, and beauty products, hoping that they are not harmful to health and reduce pollution. Moreover, the repeated mini-lock downs during the COVID-19 pandemic have fueled the awareness that body beauty is linked to well-being, both external and internal. As a result, consumer preferences for makeup have declined, while those for skincare products have increased. Nutricosmetics, which combines the benefits derived from food supplementation with the advantages of cosmetic treatments to improve the beauty of our body, respond to the new market demands. Food chemistry and cosmetic chemistry come together to promote both inside and outside well-being. A nutricosmetic optimizes the intake of nutritional microelements to meet the needs of the skin and skin appendages, improving their conditions and delaying aging, thus helping to protect the skin from the aging action of environmental factors. Numerous studies in the literature show a significant correlation between the adequate intake of these supplements, improved skin quality (both aesthetic and histological), and the acceleration of wound-healing. This review revised the main foods and bioactive molecules used in nutricosmetic formulations, their cosmetic effects, and the analytical techniques that allow the dosage of the active ingredients in the food.

## 1. Introduction

In 2020, the beauty and skincare sector had to reinvent itself to respond quickly to the new needs and requests of an unpredictable and attentive market. The most significant challenge was (and is) to find a point balance between the “natural” and the “cosmetic product’s chemistry”. Some certainties emerge regarding trends and related sectors in this fluid context, showing positive signs of recovery. The future keywords of the cosmetics sector are “sustainability” (18.9% in 2020 compared to 13.2% in 2018, based on the answers of the interviewed sample), “natural/organic” (10.9%), “care” (7.8%), “ethics” (7.5%), “e-commerce” (7.1%), “social beauty” (7.0%), “personalization” (6.7%), and “safety” (6.3%) [1]. A cosmetic can be considered “green” if its formulation contains active ingredients derived from plants, such as minerals and plants, and not analogous active ingredients chemically reproduced in the laboratory. It is better if it is produced in an eco-sustainable way through processing methods that respect nature and plants according to organic crops. It is advisable to cultivate these cosmetics at zero km or on land near the production laboratories or travel with sustainable means of transport to reduce the environmental impact. Not all green products are the same. It is necessary to distinguish between natural ingredients, natural origin, and organic ingredients. Natural ingredients are chemical substances that are unprocessed or processed by mechanical, manual, naturally derived solvent, or gravitational means, dissolution in water, heating to remove water, extracted from the air by any means. Naturally derived ingredients are substances from the vegetable, mineral, or animal kingdom, chemically processed, or combined with other ingredients, excluding petroleum and fossil fuel-derived ingredients, ingredients derived from a plant feedstock, and bio-manufactured using saponification, fermentation, condensation, or esterification to enhance performance or make the ingredient sustainable. According to the USDA National Organic Program (NOP) guidelines, organic ingredients are substances obtained by mechanical, physical, or biologically based farming methods to the fullest extent possible [2]. Well, chaos reigns over natural cosmetics in the USA and Europe, because currently there is still no official regulation that has a precise definition on how to apply the words “organic” and “natural” to cosmetic products. The United States Department of Agriculture regulates “organic”. The National Organic Program (NOP), a part of USDA’s Agricultural Marketing Service, certified organic products. Therefore, only cosmetics that contain or are made up of agricultural ingredients and can meet the USDA/NOP organic production may be certified under the NOP regulations [2]. Four categories can be applied to certified organic products, including certified organic cosmetics: 100 percent organic (they are produced with 100% ingredients certified organic); organic (they can contain up to a maximum of 5% of non-organic products, excluding water and salt); “made with” (they are produced with least 70% ingredients certified organic, excluding water and salt); and specific organic ingredients (they contain a combination of organic and non-organic substances) [3]. In Europe, this market is regulated by the ISO (International Organization for Standardization) issued ISO 16128 (November 2016) [4], a new set of guidelines for any product on the European market that claims to be natural/organic, the E.U. Regulations EC 1223/2009 [5] and EU 655/2013 [6], which requires that every declaration on a label must be supported by adequate and verifiable evidence.

In recent years, new trends have been created in the field of green cosmetics: nutricosmetics, a food supplement to use for hair, skin, and nails to obtain beauty from within. Nutricosmetic products, or so-called “*beauty supplements*”, result from the scientific work of three research areas: food, pharmaceuticals, and personal care. They are soft or hard gels, capsules, tablets, syrups, gummies, or sachets containing a concentrated source of hyaluronic acid, minerals, vitamins, or botanical extracts, able to improve personal care [7]. There is no specific regulatory framework addressing nutricosmetics at the EU and USA levels. However, the rules on food supplements govern beauty supplements [7]. In this work, the food matrix of cosmetic relevance, bioactive molecules usable in cosmetic formulations, eco-friendly technology to produce bioactive cosmetic ingredients, and the analytical techniques helpful in purifying and dosing the active ingredients in vegetable and animal matrices, are revised. We aim to shed light on the nutricosmetic market waiting for a specific regulation for green cosmetics to help consumers make informed choices.

## 2. Plant Cell Culture Technology

The growth in consumers’ interest in natural products determined the use of extracts from aromatic, herbal, and medicinal plants as active ingredients in cosmeceuticals and nutricosmetics formulations. They contain biologically active molecules (e.g., phenolic acids, polyphenols, triterpenes, stilbenes, flavonoids, steroids, steroidal saponins, carotenoids, sterols, fatty acids, sugars, polysaccharides, peptides, etc.) [8], whose profile and level depending on the pedoclimatic condition and agriculture practice [9,10]. Bioactive extracts are also obtained by algae, mushrooms, by-products of plant origins [11,12,13,14], and plant cell culture technology [15,16]. The latter is a natural and suitable technology used to make hair care, makeup, skincare, and supplement ingredients. The explant is the vegetable tissue used to start a cell culture. The cells on the surface of the explant grow in volume, divide, dedifferentiate, and form a mass called calluses. In vitro, the callus could be sustained for unlimited time using the correct growth medium. In a liquid medium, cells constitute a rapidly growing suspended culture of individual cells or small groups of cells [17]. Plant cell culture consent to produce high-value ingredients (primary and secondary metabolites) under controlled conditions. They have the advantage of maturing into a whole plant via embryogenesis, reproducing by using bioreactors independently on management practices and soil and climate conditions, producing high level of phytochemicals since some biomass in a short period are yield [18], and supplying contamination-free biomass [19]. The cosmetic extracts from plant cell cultures meet the safety requirements of the market since they are free of pathogens, pollutants, and agrochemical residues, which often contaminate plant extracts, and rarely contain toxic compound and potential allergens from plants synthesizing them to defend themselves against the attack of pathogens and pests [20]. 

## 3. Natural Antiaging 

Natural antiaging ingredients include barrier repair, moisturizing, anti-inflammatory, skin lightening, and sunblock agent.

### 3.1. Moisturizing Agents

The skin moisturizing agents can be emollients, occlusives, and humectants. 

Emollients cover the skin with a protective film to hydrate and soothe it. They contribute to decreasing flaky skin and roughness. Foods used as emollients include butter and oils such as the butter of shea, cocoa, cupuacu, mango, kombo, and murumuru butter; and the oil of almond, avocado, argan, borage, olive, babassu, broccoli, rapeseed, chia seed, castor bean, coconut, primrose, palm, passion fruit, pomegranate, raspberry, safflower, and sunflower. 

Occlusives form an epidermal barrier to stop trans-epidermal water loss and regulate keratinocyte proliferation [21]. Foods used as occlusive moisturizing agents are oils and waxes such as olive, jojoba, and coconut oils; and the wax of candelilla and bees [22]. The oils of coconut and castor have both functions as emollients and occlusives. 

Humectants are water-loving moisturizing agents that draw moisture from the dermis to the stratum corneum and binding water vapor from the environment [23]. Honey, hyaluronic acid, sorbitol, glycerine, and glycerol are examples of humectants’ moisturizing agents [24]. 

### 3.2. Barrier Repair Agents

The skin barrier stops transepidermal water loss and defends against pathogens [25]. Barrier repair agents are the essential fatty acids, phenolic compounds, tocopherols, phospholipids, cholesterol, and ceramide. The ratio of the essential fatty acids is a critical point to benefit barrier repair. Higher levels of linoleic acid to oleic acid have better skin-barrier potential [26]. It enhances the permeability of the skin barrier [26,27], being an integral component of the lipid matrix of the stratum corneum [28]. Oleic acid, disrupting the skin barrier, acts as permeability enhancers for the other bioactive molecules present in plant oils [29]. The antioxidant compounds (tocopherols and phenolics) modulate skin barrier homeostasis, wound healing, and inflammation [30,31]. Phospholipids act as chemical permeability enhancers [32]. They show anti-inflammatory effects by controlling the covalently bound, ω-hydroxy ceramides and inhibiting thymic stromal lymphopoietin and chemokine [33]. Cholesterol and ceramides are other important lipid classes in the stratum corneum [34]. Cholesterol in the plasma membrane can be an essential factor for the magnitude of the oxygen gradient observed across the cell membrane [35]. Twelve ceramide subclasses are identified in the stratum corneum [36]. Ceramide influences firm and plump skin. Topical application of a ceramide cream decreases IL-31 and damages the skin barrier’s physical and function [37]. Some natural oils contain fatty acids that play critical roles in maintaining the skin barrier. Flaxseed oil, walnut oil, and chia oil contain omega-3s and grapeseed oil, safflower oil, sunflower oil, blackcurrant seed oil, evening primrose oil, and borage oil hold omega-6s [34].

### 3.3. Skin Lightening Agents

Skin lightening agents decrease the concentration of melanin (skin’s pigment). The skin tone is lighter when there is less melanin. Skin whitening agents act as inhibitors of the tyrosinase (a key enzyme in melanogenesis) and/or melanosome transfer (pigment granules in the melanocytes, contained in the basal layer of skin epidermis) [38,39] or increasing the epidermal turnover and the effect of anti-inflammatory and antioxidant actives [40]. Ethnic differences, chronic inflammation, hormonal changes, and UV exposure are examples of conditions that can determine hypo- or hyper-pigmentation [41]. The commonly used active ingredients include citrus extracts, kojic acid, licorice extract, white mulberry extract, bearberry extract, Indian gooseberry, vitamin C, vitamin B3, hydroquinone, retinoids, resveratrol, and alpha- and beta-hydroxy acids [42]. 

### 3.4. Anti-Inflammatory Ingredients

Exogenous stimuli sometimes can determine wound, skin aging, inflammatory dermatoses, or skin carcinogenesis. Damages of the skin barrier determine the inflammatory response, which provides tissue repair and infection control. Initially, the keratinocytes and the innate immune cells (e.g., leukocytes, dendritic cells, and mast cells) are activated [43], and successively make cytokines (e.g., IL-1α, IL-6, and TNF-α) that draw the immune cells to the injury site. Finally, ROS, elastases, and proteinases are produced [43]. Thus, inflammation is involved in acne’s pathogenesis and determines pain, swelling, and redness in the skin. Licorice root, turmeric, oats, chamomile, and nuts are some food plants with anti-inflammatory activity [44,45].

### 3.5. Sunblock Ingredients

UV radiation is divided into three main categories: UV-A (320–400 nm), UV-B (280–320 nm), and UV-C (100–280 nm), based on the wavelength. Elevated exposure to UV radiation can cause edema, erythema, hyperpigmentation, photoaging, immune suppression, and skin cancer based on the intensity and range of UV radiation [46,47]. Continuous exposure to UV radiation can cause pigmentation, lesions, sunburn, dark spots, degradation of collagen fibers, wrinkles photoaging, and cancer [48,49]. UV-A photons cause damage to fibroblasts and keratinocytes [50]. In the skin, cellular chromophores absorb them, and reactive oxygen species (e.g., superoxide, hydrogen peroxide, and hydroxyl radicals) are made [51]. Oxidative stress can cause DNA damage [52]. UV-B is known as burning rays and is considered the most active constituent of solar radiation. It can induce direct and indirect adverse effects on DNA and proteins [53], inducing immunosuppression and skin cancer [54]. The most dangerous UV wavelengths are UV-C. Fortunately, these radiations are absorbed by the atmosphere before they reach our skin [55]. They are potent mutagens and can trigger cancer and immune-mediated disease [56]. *Aloe vera*, green tea, coconut oil, grape seeds, and ginger contain phytochemicals that prevent photoaging and skin cancer [24].

## 4. Skin Antioxidant Systems

Reactive oxygen species (ROS) are atoms or molecules whose last electronic layer contains unpaired electrons and molecules of excited oxygen. These agents are highly reactive and have short lives, as they react in the medium in which they are made. Molecular oxygen, hydrogen peroxide, and singlet oxygen are not free radicals but start oxidative reactions and make free radicals. Together, these species are defined as ROS. The human metabolism produces them and reactive nitrogen species (RNS) [57]. The free radicals react with other radicals, indirect iron-sulfur proteins, and transition metals (e.g., iron and copper), inducing hydroxyl formation. Hydrogen peroxide is not very reactive but can pass through membranes and react with transition metals to make the hydroxyl radical (Fenton reaction) [58]. The hydroxyl radical produces some harmful effects on the body, and the extremely short half-life makes it challenging to capture in vivo. It may attack other molecules to capture hydrogen and react with compounds by adding or transferring its electrons [59]. Lipids, proteins, and DNA are the molecules most subjected to oxidative damage. The oxidation of amino acids determines protein fragmentation, aggregation, and proteolytic digestion (no repair mechanisms for these changes). When ROS attack enzymes, our body inactivates their functions. When ROS attack polyunsaturated fatty acids (lipid peroxidation), they determine changes in membrane fluidity, constitution, selectivity, and transepidermal water loss, resulting in skin dryness. Additionally, the lipid peroxidation process enhances the expression of cyclooxygenase, phospholipases, and the production of prostaglandins, which cause epithelial inflammation [60,61]. When ROS oxidizes low-density lipoprotein (LDL), the ox-LDLs release tumor necrosis factor-α, interleukin-6, and nitric oxide, determining atherosclerosis [62]. When ROSs attack the nucleic acids, they determine mutagenesis, carcinogenesis, and aging. Our body intervenes to repair the nucleic acids by complex mechanisms rarely [63,64,65]. Some hydroxyl radicals, peroxyl, superoxide, hydrogen peroxide, and oxygen singlet are made in the skin [58]. Therefore, they can be used as indicators to assess the degree of inflammation. When the skin is exposed to free radicals, it reduces the production of ROS by suppressing the enzyme activity, which indirectly generates oxygen metabolites, increases the production of DNA repair enzymes, makes the molecules able to help the physical protection of the skin (by enhancing the stability of the membrane), and interferes with biological targets of ROS [66]. Skin cells are protected from free radicals by antioxidants such as vitamins (e.g., E, C, and A), carotenoids, ubiquinone, uric acid, hormones (e.g., estradiol and estrogen), lipoic acid, and enzymes (e.g., catalase, superoxide dismutase, and glutathione) [67]. Antioxidant molecules prevent free radicals (ROS) from oxidizing or reduce the formation or quench the formed ROS [67]. Vitamin C, alpha-tocopherol (vitamin E and derivatives), glutathione, ubiquinone are examples of primary antioxidant molecules (or free radical scavenging antioxidants). The primary antioxidant molecules decrease oxidation via chain-terminating reactions by transferring a proton to the free radical species [68]. Lipoic acid and *N*-acetyl cysteine are examples of secondary antioxidants. They reduce primary antioxidants by acting as a cofactor for several enzyme systems. Additionally, metal-chelating agents are considered secondary antioxidants because they neutralize transition metals’ production of free radicals in the skin. Often, secondary antioxidants are used in combination with primary antioxidants to protect primary antioxidants from degradation [69]. The glutathione hormone (GSH) reductase, GSH peroxidases, glutathione S-transferases (GSTs) are examples of antioxidant enzyme systems that directly neutralize ROS with the help of metal cofactors (e.g., Cu, Zn, Mn, and Se) [70]. The antioxidants found in the skin show a gradient in the human epidermis (elevated levels in the basal layers and low levels in the upper layers). The antioxidant molecules’ concentration and enzymes are decreased by intrinsic (age) and extrinsic factors (atmospheric components). Sunlight (in particular solar ultraviolet radiation UVA and UVB) causes ROS generation in the skin. UVB radiations enhance the production of O_2_^−^ by activating NADPH oxidase and the reaction of the respiratory chain [71,72], improving the expression of nitric oxide synthase, the production of highly reactive anion peroxynitrite, of the melanin by melanocytes, and the expression of metalloproteinases (enzymes able to degrade collagen) [70]. UVA radiations produce ^1^O_2_ by photosensitizing internal chromophores (e.g., porphyrin and riboflavin), and glycation products [73], and activating NADPH oxidase [74]. UVB radiations induce erythema (improving prostaglandin E2 synthesis) [75], skin roughness (oxidizing the lipids) [76], enhance the production of the carbonylated proteins in the stratum corneum (SCCP), and stimulate sebum secretion [77]. Therefore, it is clear that it is worth replenishing antioxidants through topical application or dietary supplements to protect the skin [78,79].

## 5. Methods for Determining the Antioxidant Activity of a Natural Extract

Chemical-based and cellular-based assays can evaluate the antioxidant potential of a natural extract. Chemical-based methods measure single electron transfer (SET assay) or hydrogen transfer (HAT assay) (e.g., ORAC, TRAP). SET methods can scavenge free radicals (e.g., DPPH) or reduce metal ions (e.g., FRAP, CUPRAC) [80,81,82]. It is necessary to use both methods (SET and HAT) for the correct evaluation of the total antioxidant activity [83,84,85] since, in a natural extract, there may be more than one class of molecules capable of carrying out this activity.

### 5.1. Methods Used to Determine the Antioxidant Potential

#### 5.1.1. Spectroscopic Methods

##### Trolox Equivalent Antioxidant Capacity (TEAC) Test

The TEAC is a free radicals scavenging method. It evaluates the ability to scavenge the ABTS radical [86]. It is possible to use two different oxidizing agents to obtain the goals: metmyoglobin-H_2_O_2_ or potassium persulfate. Both agents oxidize the ABTS, making ABTS^•+^ (colored), then the addition of antioxidants causes a loss of the green color spectrophotometrically evaluable (λ 734 nm) [78,85]. This method detects the antioxidant potential of lipophilic and hydrophilic extracts and is not affected by ionic strength [85]. Briefly, K_2_S_2_O_8_ (3 mM) react for 16 h with ABTS dissolved in distilled water (8 mM) in the dark at room temperature. Then, the ABTS^•+^ solution is diluted in phosphate buffer solution (pH 7.4) and NaCl (in PBS 150 mM). The absorbance of 1.5 at 730 nm is read. Reaction kinetics are performed by taking readings every 15 min over a 2 h period. The reaction time is determined (generally 30 min.). Standards (100 μm) and samples (100 μm) are reacted with ABTS^•+^ (2900 μm) for the reaction time previously determined [85]. The antioxidant potential was expressed as Trolox equivalents [85].

##### 2,2-Diphenyl-1-picrylhydrazyl (DPPH) Test

The DPPH detects the ability of a compound to transfer one electron [79]. The antioxidants reduce DPPH radical to DPPH-H [79]. The decrease of the absorbance value at λ 515 nm (DPPH absorbance) indicates the antioxidant potential. This test overestimates antioxidants with many phenol groups as flavonols [86]. Briefly, samples (20 μL) are added to 3 mL of DPPH solution (6 × 10^−5^ mol/L), and the spectrophotometric analysis is performed. The absorbance is read at λ 517 nm every 5 min until the steady state. The calibration curve is made using 6-hydroxy-2,5,7,8-tetramethylchroman-2-carboxylic acid (Trolox). The results were expressed as mmol Trolox equivalent (TE) kg^−1^ FW [87]. 

##### The Ferric-Reducing Antioxidant Power (FRAP) Test

The FRAP assay measures antioxidants’ ability to reduce a ferric tripyridyltriazine (Fe^3+^-TPTZ) to the ferrous (Fe^2+^-TPTZ). The antioxidant’s power is positively related to absorbance absorption at λ 593 nm. [87]. FRAP cannot detect proteins and thiols which have radical-quenching abilities. This test work at pH 3.6 [79]. Briefly, a solution of TPTZ (10 mmol/L) is added in HCl (40 mmol/L), ferric chloride (12 mmol/L), and sodium acetate buffer (300 mmol/L, pH 3.6) at a ratio of 1:1:10. Samples and standard antioxidant solutions (both 1 mmol/L) are added to the FRAP solution (3 mL). They must react for 90 min at 37 °C before taking the spectrophotometric reading at λ 593 nm [87].

##### The Cupric-Reducing Antioxidant Capacity (CUPRAC) Test

The CUPRAC assay measures antioxidants’ ability to reduce Cu(II)-neocuproine (Nc) at λ 450 nm after 30 min. [88]. This test works at pH 7, detects the antioxidant potential of both lipophilic and hydrophilic antioxidants [88], and determines the reducing power of thiol-type antioxidants [89]. Briefly, sample (0.1 mL;) is mixed with distilled water (1 mL) copper chloride (0.4262 g dissolved in H_2_O and diluted to 250 mL with additional water), neocuproine (7.5 × 10^−3^ M), and ammonium acetate buffer solution (19.27 g in water and diluting to 250 mL; pH 7) at 1:1:1 to obtain total reaction mixture of 4.1 mL. They must react 30 min at room temperature before taking the spectrophotometric reading at λ 450 nm. Results were expressed as μM Trolox equivalents [89].

#### 5.1.2. In Vitro Cellular Methods

##### The Dichlorofluorecin (DCFH) Test

The DCFH test assay measures antioxidants’ ability to prevent the oxidation of dichlorofluorecin into dichlorofluorescein (DCF) by 2,2’-azobis (2-amidinopropane) (ABAP)-generated peroxyl radicals in human hepatocarcinoma cells (HepG2 cells). Antioxidant power is negatively related to cellular fluorescence growth (λexc = 485 nm, λem = 538 nm) [90]. Briefly, myelomonocytic cells (HL-60, 1 × 10^6^ cells/mL) are suspended in Roswell Park Memorial Institute (RPMI 1640) medium with 10% fetal bovine serum (FBS) and antibiotics in 5% CO_2_: 95% air at 37 °C. The cell suspension (125 μL) is added to the plates, treated for 30 min with the test material, and stimulated with phorbol 12-myristate 13-acetate (PMA,100 ng/mL) 30 min. Then, the cells are added to molecular probes (5 μg/mL DCFH-DA) and incubated for 15 min. DCFH-DA is a nonfluorescent probe that diffuses into cells. The levels of DCF are measured using a fluorescence measurement system [90].

##### Determination of the Lipid Peroxide Levels

The detection of lipid peroxidation can evaluate in skin keratinocytes cells (HaCaT cells). Epinephrine is used to induce lipid peroxidation. Antioxidant ability is negatively related to cellular fluorescence growth (λexc = 510 nm, λem = 580 nm) [16]. Briefly, HaCaT (1.8 × 10^4^) are seeded in 96-well plates and then incubated for 24 h with samples or beta-blocker (ICI-118,551). Successively, the cells are washed in phosphate-buffered saline (PBS) and then incubated for 30 min with the lipid peroxidation sensor (dyeC11-Bodipy) at 37 °C. Finally, epinephrine (50 μM) is used to make lipid peroxidation. The levels of peroxidized lipids are measured using a fluorescence measurement system [16].

##### Determination of the Carbonylated Protein Levels in Cells

The carbonylated protein levels can be evaluated in a spontaneously transformed aneuploid immortal keratinocyte cell line from adult human skin (HaCaT cells) via enzyme-linked immunosorbent assay (ELISA) using a specific antibody against 2,4-dinitrophenol (DNP). Epinephrine is used to induce protein carbonylation. The ability of antioxidants is negatively related to the growth in cellular fluorescence [16]. Briefly, HaCaT (1.5 × 10^4^) cells were put in 96-well plates and incubated for 24 h with the samples or ICI-118,551 and epinephrine (50 μM). Successively, the cells were washed in PBS and fixed in 4% paraformaldehyde (PFA). Then, cells were washed with PBS and 0.05% Polyethylene glycol sorbitan monolaurate (Tween 20) and incubated for 1 h with 2,4-Dinitrophenyl-hydrazine (DNPH; 5 mM) in 2 N HCl at room temperature. The carbonylated products were detected using a specific antibody against DNP (sc69697) by using the ELISA method. The skin punches were incubated for 24 h with the sample and epinephrine (56 nM). Successively, the punches were fixed for 6 h with PFA, washed in PBS, incubated in sucrose (15% and 30%), fixed in optimal cutting temperature (OCT) medium, frozen, and stored −80 °C. Cryosections (10 μm) were incubated with DNPH (5 mM) in 2N HCl for 1 h at room temperature, washed in PBS/EtOH (1:1), and PBS/Tween 20. The slides were incubated for 30 min in BSA, washed with PBS/Tween 20, incubated with antibody anti-DNP (1:50 dilution), and then mixed with the conjugated antibody Alexa Fluor 488. The signals were measured using fluorescent microscopy [16].

## 6. In Vitro Methods for Determining Antioxidant Bioaccessibility

Bioaccessibility shows the concentration of antioxidants potentially available for absorption after each step of digestion. It is an essential condition to know bioavailability [91]. Some in vitro tests show the antioxidant levels available for physiological functions.

### 6.1. Gastric Digestion Simulation

The gastric digestion reproduction is found, including artificial saliva and pepsin, into samples at pH 2 (gastric pH). Briefly, samples are incubated at 37 °C for 2 h with artificial saliva (6 mL), pepsin (14,800 U; 0.5 g), HCl (0.1 N), and blended in an orbital shaker at 55 rpm [91]. The acidification of the samples prevents the denaturation of pepsin that occurs at pH ≥ 5 [91].

### 6.2. Intestinal Digestion Simulation

The intestinal digestion is obtained, including bile salts and pancreatin at pH 5.5–6, and the pH at 6.5 value is readjusted [91]. Briefly, the sample pH is adjusted to 6.5 with NaHCO_3_ (1 N) and successively mixed with pancreatin (8 mg/mL; 1:1; *v*/*v*), bile salts (50 mg/mL), and 20 mL water. Successively, the solution is incubated for 2 h at 37 °C and blended in an orbital shaker at 55 rpm. Finally, the solution (30 mL) is centrifuged (4000× *g* rpm at 4 °C) for 1 h. The supernatant (bio-accessible fraction) was collected and monitored using spectrophotometric methods [91].

## 7. In Vivo Method to Evaluate the Oxidative Damage in the Skin

Vertuani and colleagues proposed an in vivo protocol to evaluate oxidative skin damages [92]. In this model, methyl nicotinate (M.N.) is used to enhance the prostaglandins and cyclooxygenase synthesis (an inflammatory process), and antioxidant potential is evaluated by measuring:

- Transepidermal water loss to determine the barrier function of the stratum corneum, by Tewameter TM 210 (Courage-khazaka, Cologne, Germany);
- The skin color of the sites before and after the irritation by using a reflectance meters Chromameter (CR-300 Minolta);
- The cutaneous microcirculation by using a Laser Doppler Perfusion Imager (PIM1.0 Lisca Development AB, Sweden);
- The color analysis of derma by DermAnalyzer^®^, a new software program, developed by Manfredini and colleagues, using the CIE L*a*b* color-space parameters (the color space specified by the French Commission Internationale de l’éclairage, hence its CIE initialism) [92].

Briefly, the study is conducted on two homogenous healthy groups of volunteers, one treated with the product to be tested, the other with placebo. The measures are performed before stress, acute stress, and after the recovery time. The measures are performed in a closed room (temperature = 20 ± 2 °C; relative humidity = 40 ± 5%) at the same time of day after a stationing time of about 30 min. The probes are calibrated before the measures. Then, they are applied to the skin for 30 s to obtain the measures. Tewameter probe measures the water evaporation rate (g/h/m^2^). Chromameter measures color quality with CIE L*a*b* and CIE L*C*h color spaces in numbers to communicate colors precisely. Laser Doppler Perfusion Image measures microcirculation using a laser beam to scan tissue and a photodiode to detect the reflected light. The processed signal generates an image that shows the perfusion in the tissue [92]. 

## 8. Phytochemicals and Vitamins with Antioxidant Potential

The secondary metabolism of plants makes molecules (phytochemicals) that can defend plants from the attack of atmospheric agents, microbes, and pests. Some of these (e.g., phenolic acid, polyphenols, cysteine sulphoxides, carotenoids) can react with free radicals to form stable chemical species in humans and animals [93]. Phytochemicals have a wide range of biological effects (e.g., photoprotective, antiaging, anti-inflammatory, antibacterial, antiviral, and anticancer effects) beneficial for our wellbeing [93]. Some vitamins (e.g., E, C, and A) have antioxidant potential and skincare abilities. Vitamin C regulates collagen synthesis. Vitamin E is active in neutralizing the free radicals and softening the skin [94]. Vitamin A controls the production of new skin cells and increases collagen production, minimizing burns, scars, and stretch marks [95]. 

### 8.1. Analytical Procedure for the Quantification of Antioxidants in Food Extract

Several analytical methods have been proposed in the literature to determine the single antioxidant compounds in natural extracts. However, many tests, although used routinely, lack a robust validation procedure. It would be necessary to validate analytical methods to allow correct dosage and data traceability, waiting for stringent legislation on the use of active ingredients of natural origin in cosmetics [96]. The analytical procedures for quantifying antioxidants involve two basic steps: extraction from the organic matrix and quantification.

#### 8.1.1. Phenolics

The plant phenols have a benzene ring with a hydroxyl group attached and some substituents (e.g., ester and glycosides). Some plant phenols have more than one hydroxyl group. The phenol classification is carried out based on the number of phenolic rings and the number and type of substituents present on the phenolic rings [97]. Examples of simple phenols are phenolic acids (e.g., gallic and ferulic acids). Examples of polyphenols are stilbenes (e.g., resveratrol), chalcones, and flavonoids. Flavonoids are further divided into flavonols, flavanols, flavones, flavanones, flavanonols, isoflavones, and anthocyanins [98]. Phenols have skin-healing and protective effects [99]. Theaflavins prevent Herpes Simplex type I (HSV-1) and protect against UV-induced photoaging and photo immunosuppression [100]. Anthocyanins reduce skin damage due to solar radiation [101], decreasing UVA-stimulated ROS formation, lipid peroxidation [102], and modulating NF-kB- and MAPK-dependent pathways responsible for the inflammatory response [103]. The phenolic compounds isolated from *Malus dounteri* A. Chev. have anti-elastase and anti-MMP-1 activity in human skin fibroblast cells [104]. The aldehyde polycondensates of (β)-catechin have anti-elastase and anti-collagenase activities [105]. The pistachio nuts’ polyphenol decreases UVB-induced skin erythema. Oral consumption of polyphenols decreases skin roughness and improves skin hydration and elasticity. The combination therapy of topical application and oral intake enhances the results [106].

##### Total Phenolics Extraction Method

Today, some methods are proposed to extract phenolics, among these: solid extraction (SE), ultrasonic, microwave-assisted extraction (MAE), molecularly imprinted polymers, solid-phase extraction (SPE), pressurized liquid extraction (PLE), enzyme-assisted extraction (EAE), and supercritical fluid extraction (SFE) [107]. The SPE methods are preferred since they are easy to use and consume a short extraction time. Several types of stationary phases are employed (e.g., C8 cartridges, amino-phase cartridges, diol-bonded phase cartridges, octadecyl C18, and octadecyl C18 end-capped) [108].

##### Total Phenolics Dosage Method: Folin–Ciocalteu Test

Folin–Ciocalteu is a colorimetric assay based on the reaction of Folin–Ciocalteu reagent with the hydroxy groups of phenolics. Polyphenol levels are positively related to absorbance growth (λ 765 nm) [109].

#### 8.1.2. Carotenoids

The principal carotenoids’ class of compounds are xanthophylls and carotenes. Carotenes are strictly hydrophobic molecules. Instead, xanthophylls have polar groups in their structures. Then, there are strict hydrocarbon carotenoids (e.g., lycopene and β-carotene) that do not have any substituent in their structures, some with epoxy groups (e.g., diadinoxanthin, violaxanthin), others with acetyl groups (e.g., fucoxanthin, dinoxanthin), and finally some with acetylene (e.g., diato-, allo-, diadino-, pyro-, croco-, hetero-, and monadoxanthin). Carotenoids in the skin have an essential role in photoprotection against UV radiation since they have antioxidant and anti-inflammatory actions. Astaxanthin enhances superoxide dismutase, catalases enzyme activities [110], and suppresses tyrosinase activity [111]. Its oral use improves skin condition and decreases skin hyper-pigmentation and melanin synthesis [112]. β-carotene prevents free radicals formation. It inhibits wrinkle formation and skin sagging, decreasing metalloproteinase-9 activation and improving 5-α-hydroperoxide synthesis, and protects from sunburn diseases [113]. The β-carotene has a skin photoprotection effect more homogenous when it is orally supplemented than topically applied even if its protection factor varies in the two forms of application [104]. Orally consumed beta-carotene has a sun protection factor (SPF) 4. Instead, the SPF of beta-carotene topically applied is from 10 to 40 [114]. The supplements of lycopene decrease skin roughness [115]; of the xanthophylls, increases skin hydration [116]; of the lutein, protect skin from skin damage and photoaging [116]. Oral and topical treatment with zeaxanthin and lutein improves skin layer hydration and skin elasticity [117] and defends the skin against oxidative damage and blue-light damages [118]. Lutein decreases lipid peroxidation in the cell membrane and scavenges free radicals [114]. Lycopene enhances dull skin, decreases skin roughness [119], has a sun-screening effect, and acts as a sunburn protection agent [120].

##### Carotenoids Extraction Methods

The solubility of carotenoids depends on their molecular structure (xanthophylls, carotenes). Generally, tetrahydrofuran is considered the best solvent for solubilizing carotenoids, but it is used with antioxidants (e.g., butylated hydroxytoluene -BHT) since it forms peroxides. The xanthophylls (e.g., lutein) are soluble in alcohols and carotenes (e.g., β-carotene) in hydrophobic solvents [121]. Some carotene-extraction methods employ enzymes (used to break down the plant tissue in which carotenes are located), organic solvents (e.g., hexane and ethyl acetate) since they are lipophilic compounds, and water-miscible solvents (e.g., acetone and tetrahydrofuran) to complete the penetration in the food matrix [122]. Sometimes, magnesium carbonate or calcium carbonate is added to neutralize organic acid. The extractions must be repeated until the residue and filtrate become colorless [121]. Supercritical fluid extraction (SFE) with carbon dioxide (CO_2_) is an alternative to liquid–liquid extraction methods. The SFE is a low-cost, non-toxic, and eco-compatible method since the extracts are chemical residue-free and require small amounts of organic solvents. The extraction efficiency improves with temperature and pressure [121]. Another option to extract carotenoids is the MSPD (Matrix solid-phase dispersion). In this case, the sample is extracted using a bounded-phase solid support material (e.g., C18) [121]. 

##### Carotenoids Quantification Method

The most used method to dosage the carotenoids is UV/Vis spectrophotometry. The UV/Vis spectrum offers information about the carotenoid’ chromophore, which is absorbed in the range (λ 400–500 nm) [121]. For example, the lycopene is quantified spectrophotometrically at λ 502 nm and λ 455 nm [91].

#### 8.1.3. Vitamins

The vitamins A, C, and E are used in skin aging and UV protection treatment [123]. Their esterified forms are preferred in topical formulations having more stability than free forms [124]. Retinyl palmitate has a beneficial effect on dry and rough skin epithelization and abnormal keratinization [125]. Vitamin C enhances skin hydration [126]. Tocopheryl acetate has a free radical scavenger activity, decreases DNA damage, keratinocyte death [127], skin roughness, and improves stratum corneum hydration [128]. A topical combination of vitamins C and E maximizes photoprotection [129].

##### The AOAC Method for Dosage of Vitamin A (AOAC Official Method 970.64)

The AOAC method to dosage the vitamin A recommends an extraction with acetone-hexane followed by filtration, a second extraction with water to remove acetone, and chromatography of esane extract (by using activated MgO_2_ diatomaceous earth column as stationary phase and acetone as mobile phase) combined with a colorimeter [130].

##### The Method for Dosage of Vitamin C

Vitamin C’ dosage method recommends an extraction with 3% mete-phosphoric acid-acetic acid of the food matrix, followed by oxidation with Norit of ascorbic acid into dehydroascorbic acid, and reaction with *O*-phenylenediamine to make a fluorescent derivative which is isolated by an inverse chromatography (stationary phase: 10 µm-µBondapak C18; mobile methanol: water/55:45) and detected fluorometrically [131].

##### The Method for Dosage of Vitamin E

Vitamin E’s dosage method recommends isolating α-tocopherol by extraction, followed by saponification of lipid extract and TLC chromatography, and identification by the colorimetrical procedure. To dosage a-tocopheryl acetate, the sample is extracted, natural a-tocopherol is taken off by oxidative chromatography; then, the a-tocopheryl acetate is saponified and identified colorimetrically [132].

#### 8.1.4. S-Alk(en)yl-l-cysteine Sulfoxides (ACSOs)

ACSOs have antioxidant properties. They enhance the aspartate transaminase, alanine transaminase, and lactate dehydrogenase activities and decrease thiobarbituric acid reactive substances, glutathione levels, and glutathione S-transferase and glutathione peroxidase activities [133]. The ACSOs and their transformation products have antimicrobial potential due to their antioxidant activity and inhibition of thioredoxin reductase, alcohol dehydrogenase, trypsin, RNA, and DNA polymerases [134]. *N*-acetyl-l-cysteine, in keratin molecules, can interact with disulfide bridges, causing nail swelling and softening and facilitating drug permeation [135].

##### S-Alk(en)yl-l-cysteine Sulfoxides (ACSOs) Dosage Method

The ACSOs’s dosage method recommends isolating ACSOs by extraction with methanol:chloroform: water/12:5:3 and keeping them overnight at −20 °C. Successively, the diastereomeric S-butyl-l-cysteine sulfoxide must be added as an internal standard and must be separate the phases at room temperature by centrifugation at (12,000× *g* for 5 min). Next, the upper phase must be concentrated on a rotatory evaporator at 30 °C. Finally, the extract must be resuspended in 0.03 M HCl, filtered through a 0.45-μm filter, and analyzed by HPLC (stationary phase: C18 Hypersil ODS; mobile phase 0.03 M HCl; diode array detector) [136].

#### 8.1.5. Methylxanthines

Methylxanthines (caffeine, theophylline, and theobromine) are good antioxidants [137], since there is a quenching effect on hydroxyl radicals’ production and oxidative DNA breakage by hydroxyl radicals [138]. Caffeine improves UVR-mediated skin reactions in human skin [139]. It is actives in subjects suffering from hair loss due to premature termination of the hair-growth phase [140] and enhances lipolysis and fat oxidation in cellulite cosmetic products [141]. Caffeine controls the lipolysis process regulating the catecholamine secretion, which activates β-2 adrenergic receptors, the concentration of cyclic adenosine monophosphate (cAMP) in cells that activates lipase [142], blocking α-adrenergic receptors [143,144], and inhibiting the phosphodiesterase [145].

##### Total Methylxanthines Dosage Methods

Some methods are used to isolate methylxanthines, among these: SPE (solid-phase extraction), LLE (liquid-liquid extraction), MAE (microwave-assisted extraction), UAE (ultrasound-assisted extraction), SPME (solid-phase microextraction), and SFE (supercritical fluid extraction) [146,147,148]. Water is a suitable solvent for methylxanthines, but it has low selectivity. Therefore, a second extraction involves dichloromethane or chloroform to complete the isolation. In SPE, supercritical carbon dioxide with water, methanol, ethanol, or isopropanol is used as a solvent [147]. The most common method for analyzing methylxanthines is RP-HPLC (reversed-phase high-performance liquid chromatography) using a C18 column (stationary phase) and mass spectrometry detector [149]. Paradkar and Irudayaraj (2006) described a method based on Fourier Transform Infrared (FTIR) spectroscopic as fast (5–10 min), nondestructive, and reliable for the routine dosage of the methylxanthines in foods. In this test, partial least square (PLS) and principal component regression (PCR)^2^ were employed for dosage at two spectral regions (1500–1800 cm^−1^ and 2800–3000 cm^−1^) [150].

## 9. Foods in Cosmetic Preparation

### 9.1. Green Tea

Green tea (G.T., *Camellia sinensis*) (Table 1) extracts contain catechin derivatives (e.g., epicatechin, epicatequinagalato, epigallocatechin, and epigallocatechin-3-gallate) that can scavenge free radicals. Formulations with 6% G.T. have a prolonged moisturizing effect, improve microrelief, and reduce skin roughness [151]. Topical application of G.T. prevents UV-oxidative injury, reduces the matrix metalloproteinases, collagenase, and hyaluronidase production [152,153], and decreases UV-induced erythema [154]. Tea used orally and topically decreases sebum production, and prevents and treats acne vulgaris [155]. Anti-acne activity is ascribable to antimicrobial properties against *Propionibacterium acnes*, the increase of apoptosis of the SEB-1 cell line of sebocytes, the reduction of the lipogenesis by regulation of MLPK-SREBP-1 (M locus protein kinase-Sterol regulatory element-binding protein 1), and of the inflammation by reducing NF-ĸB (nuclear factor-κB) production [155]. In addition, the use of epigallocatechin-3-gallate improves hair growth via proliferative and antiapoptotic effects on scalp follicle dermal papilla cells and prolongs the anagen stage [156].

### 9.2. Coffea arabica

*Coffea arabica* (Table 1) contains antioxidant compounds such as proanthocyanidins, quinic acid, caffeic acid, and chlorogenic acid [157], acting as skin-lightening agents, decreasing ROS formation and tyrosinase synthesis [158,159]. In addition, the use of 0.1% coffeeberry cleanser and 1% coffeeberry cream enhances wrinkle, fine line, and pigmentation in patients with actinic damage after a 6-week treatment period [160].

### 9.3. Vitis vinifera

*Vitis vinifera* (Table 1) contains resveratrol, a stilbene with antioxidant properties able to control skin cancer, UV light-mediated skin aging, and other inflammatory disorders [161]. Grape seed polyphenolic compounds (proanthocyanidins and procyanidins) have skin-lightening properties [162] since they can inhibit ROS and scavenge free radicals [157,162]. The lightning mechanism exhibited by proanthocyanidins is related to their antioxidant properties able to reduce melanin biosynthesis. The oral intake of proanthocyanidin-rich grape seed extract enhances hyperpigmentation in women with chloasma [162].

### 9.4. Pomegranate 

*Punica granatum* (pomegranate) (Table 1) contains ellagic acid, punicalagin, and punicic acid. The ellagic acid and punicalagin enhance skin health by impeding tyrosinase and promoting antifungal and anti-inflammatory effects [163,164,165]. In addition, punicic acid acts against UV-induced radiation [166]. Ellagic acid is a phenolic component approved as a lightening ingredient for cosmetic formulations since it chelates copper ions present in tyrosinase enzymes [167] and decreases UVB-induced hyperpigmentation [168]. Pomegranate also can improve the thickness, hydration, elasticity values of the dermis [169], skin wrinkling [170], and decrease glycation scavenging free radical and inhibiting fructosamine formation in the Maillard reaction [171]. In addition, skin glycation affects collagen deteriorating skin elasticity. Therefore, pomegranate extract eliminates wrinkles due to damage from UV and skin aging.

### 9.5. Soybeans

*Glycine max* (soybean) (Table 1) contains the isoflavone genistein that can reduce UV-induced oxidative DNA damage [172] and skin photodamage [173,174,175]. The isoflavones can stimulate fibroblast proliferation, decrease collagen breakdown, and impede the protein tyrosine kinase activity [176,177,178,179]. The extracts containing more than one isoflavone and aglycone form of isoflavones (unconjugated forms) have higher beneficial effects [180,181,182]. Some antiaging sunscreens and facial moisturizers contain genistein.

### 9.6. Aloe vera

*Aloe vera* (Table 1) contains aloesin [183], which produces a skin-lightening effect, inhibits melanogenesis, and decreases tyrosinase and DOPA polymerase actions [156,183,184,185]. Mucopolysaccharides and the amino acid profile of *Aloe vera* (e.g., arginine, histidine, threonine, glycine, serine, and alanine) improve water retention in the stratum corneum [186]. *Aloe* gel has antioxidant properties. It enhances the metallothionein, superoxide dismutases, and glutathione peroxidase activities in skin-cells act. *Aloe* makes the skin elastic and reduces wrinkles, improving elastin and collagen production by fibroblasts [187]. *Aloe* gel has wound-healing effects. It keeps the wound moist, reduces the inflammation process, and enhances the epithelial cell migration and rapid maturation of collagen [188]. Finally, *Aloe* gel promotes hair growth through pilosebaceous targeting in a rat model [189].

### 9.7. Citrus limon

Hesperidin (flavanone) and ascorbic acid in lemon (Table 1) can decrease tyrosinase activity and prevent melanin biosynthesis [157,183]. Additionally, citral, d-limonene, and β-pinene have a depigmenting effect. They decrease tyrosinase activity and L-dihydroxyphenylalanine (l-DOPA) oxidation [190]. Hesperidin and ascorbic acid are used in antiaging cosmetics since they are antioxidant compounds [40,191,192]. Hyalurosomes and glycerosomes carriers are used to meliorate the antioxidant potential of lemon extracts in skin-building structures [193]. Vitamin C is used in antiaging products to reduce thin wrinkles, improving collagen production [191]. Lemon-derived products positively affect acne-prone skin that is affected by mycosis and sunburn [194]. Lemon juice mixed with olive oil is used to treat scalp and hair disorders [195].

### 9.8. Opuntia ficus indica 

*Opuntia ficus indica* (Table 1) has restorative and antiaging properties for skin, hair, and nails. The high levels of linoleic acid stimulate cell renewal, favoring deep and quick penetration through dermal layers, oleic and stearic acid supporting the skin moisturizing and collagen production, whereas palmitic acid prevents wrinkles, reinforcing the skin’s barrier function [196].

### 9.9. Ficus carica

*Ficus carica* (Table 1) contains ficin and phenolic compounds that can be used to formulate skincare products for dry and stressed skin [197]. The phytochemicals contained in *Ficus carica* extracts alleviate skin damage due to stress hormone activity, such as oxidation, inflammation, skin turning to a pale color, and alteration of the skin barrier. The treatment with *Ficus carica* extract restores the regular epidermal, improves skin lightness, and reduces sebum production and exfoliation in the clinical tests [16]. A topical cream containing *Ficus carica* fruit extract can reduce hyperpigmentation, wrinkles, acne, and freckles [198].

### 9.10. Cynara scolymus

The extract of *Cynara scolymus* (Table 1) has anti-inflammatory and antioxidant properties. Furthermore, it enhances the vasodilatation and microcirculation of endothelial cells, decreases NO production, defends the lymphatic vessels from ROS formation, and improves cellular cohesion by reinforcing the tight junction complex [199]. In addition, it increases roughness and skin elasticity. Therefore, the extracts of *Cynara scolymus* in cosmetic formulations are used as a photoprotective agent and enhance roughness and skin elasticity [200].

### 9.11. Carica papaya

The *Carica papaya* (Table 1) is used in anti-skin aging cosmetics since it contains flavonoids (e.g., kaempferol, quercetin, myricetin, and their glycosides) and phenolic acids (e.g., ferulic acid, caffeic acid) [201,202] that have an antioxidant and anti-inflammatory action [203,204]. The *Carica papaya* fruit metabolites can scavenge ROS, decrease NF-κB, improve SOD and CAT activities [204], downregulate MMPs expression, and have photoprotective action against collagen degradation. Caffeic acid reduces skin erythema via inhibitory action towards NF-κB and AP-1 signaling [205]. Cysteine endopeptidases and chymopapain have proteolytic wound-debridement and antibacterial effects [206,207,208,209].

### 9.12. Glycyrrhiza glabra

*Glycyrrhiza glabra* (licorice) (Table 1) has antioxidant, anti-inflammatory, and UV protection potential [210]. It contains flavonoids (e.g., glabridin, glabrene, isoliquiritigenin, licochalcone A, and liquiritin) with depigmenting abilities and tyrosinase inhibition effects [211] used to prevent pigmentation disorders (e.g., age spots, melasma, and sites of actinic damage) [212]. In addition, the *Glycyrrhiza glabra* extract can be used as a deodorant agent since it decreases the unpleasant odors emanated from the feet, axillae, and head regions, preventing the diacetyl formation produced by resident skin bacteria [213]. Finally, the hydro-alcoholic extract of licorice improves hair growth [214].

### 9.13. Theobroma cacao

Cocoa beans (Table 1) contain polyphenols (e.g., flavan-3-ols, proanthocyanidins, anthocyanins) and methylxanthines (e.g., theobromine and caffeine) [215] that have antioxidant and antiradical properties [216,217]. Topical application of Cocoa polyphenols regulates collagen I, III, and IV and glycosaminoglycan production [216]. Their oral consumption has anti-inflammatory, antioxidant, and photoprotective [217]. The cocoa extract incorporated into microemulsion is used in skincare formulation [218].

### 9.14. Prunus dulcis (Almonds)

Almonds are rich in triterpenoids (e.g., urosolic, betulinic, and oleanolic acids), catechin, flavonol glycosides, phenolic acids (e.g., protocatechuic acid and vanillic acid), phytosterols, fatty acids, and lipid-soluble vitamins [219,220]. The *Prunus dulcis* extract has antioxidant properties [221]. It can be used to treat eczema and pimples [222]. The almond oil nourishes, softens, and strengthens the hair [223].

### 9.15. Coconut

Coconut oils have oxidative stability ascribable to high contents of saturated fatty acids (e.g., myristic, lauric, and palmitic acids) [224]. Coconut oil protects our skin from UV rays. It can block 20% of UV rays [225]. Coconut milk softens the skin and removes black spots on the face because it is rich in natural fatty acids and contains antiseptics [226]. Consumption of coconut oil has potent anti-inflammatory effects [227]. Topic application of coconut oil on the limbs can moisturize skin [228]. Instead, it reduces protein loss if put to the hair before or after shampooing [229]. Coconut oil can be used as natural deodorant [228], body scrub, lip scrub, shaving cream, and personal cleansing agents (e.g., soaps, shampoo, and detergents) [229,230,231,232].

## 10. Strategies in the Delivery of Natural Products in Cosmetic Formulations

Many natural products with cosmetic potential are unable to penetrate the skin, are unstable to the environment, degrade in the gastric, are poorly bioavailable and soluble, and have a rapid metabolism and uncontrolled-release; therefore, they cannot be used in cosmetic formulations because they are unable to carry out their biological activity [233]. Some delivery systems are used to solve this problem. Among these, food-grade materials from proteins (e.g., whey proteins, gelatins, caseins, cereal proteins, soy proteins, and pulse proteins), lipids, and polysaccharides (e.g., starch, pectins, cellulose, alginate, chitosan, and gums) are employed due to their safety and biodegradability. For example, pomegranate bioactive compounds are added in several nanostructures (e.g., nanoemulsion, phytosomes, nanoliposomes, nanoparticles, niosomes, and nanovesicles) to be transported to the site’s action [234]. 

### 10.1. Lipid-Based Nano-Encapsulation Systems

The lipid-based nano-encapsulation systems are broadly used since they are stable, control release, and sustain release profiles [235].

#### 10.1.1. Liposomes

The liposomes are cell-like spherical bilayer vesicles with unilamellar or multilamellar structures that can protect and encapsulate lipophilic and hydrophilic compounds. They are generally made with phosphatidylcholine [235,236,237,238] and have a hydrophobic tail and hydrophilic head [239]. They can have a variable size (from 20 nm to several micrometers) [238]. Vitamins (e.g., A, E, and K) and antioxidants (e.g., CoQ10, carotenoids, and lycopene) are included in liposomes to improve their chemical and physical stability when they are dispersed in water [240]. At 4–25 °C, the stability of liposomes in an aqueous or hydroalcoholic jelly environment varies from 2–3 years. Liposomes made by polymerization of phospholipids covered by a mixture of polysaccharide and collagen, γ-globulin, or albumin are also stable [241]. Liposome stability is preserved by employing phospholipids with saturated acyl chains (e.g., hydrogenated soybean) to prevent oxidation and avoid the hydrolysis of the ester groups at pH values near the 4.5–6.5 or dispersing liposomes in a lipid solution with surfactant [242]. Some specialized liposomes are made with enzymes such as ultrasomes (they contains an enzyme extracted from *Micrococcus luteus* that can recognize sun damage and remove the damaged DNA), and photosomes (they contains a photo-reactivating enzyme extracted from a marine plant that can protect from sunlight injuries) [241]. 

#### 10.1.2. Niosomes

Niosomes are cell-like spherical bilayer nano-vesicles with unilamellar or multilamellar structures. They are made up of self-assembly of hydrated nonionic surfactants (e.g., spans, brijs, tweens, sorbitan ester, alkyl amides, crown ester, steroid-linked surfactants, and polyoxyethylene alkyl ether), with or without cholesterol or lipids [243]. Their size ranges vary from 100 nm to 2 μm [244]. Numerous moisturizing, anti-wrinkle, skin-whitening creams, conditioners, and hair-repairing shampoos are formulated with noisome [245,246].

### 10.2. Nanoemulsions

The nanoemulsions are a dispersion of liquids in which a surfactant combines the oil phase and water phase stably. There are three types of nanoemulsions (water in oil, oil in water, and bicontinuous nanoemulsion) with variable sizes from 50 nm to 200 nm. They generally have low viscosity, high interfacial area, high solubilization capacity, and high kinetic stability [247]. In cosmetics, nanoemulsions are used to make available rapid penetration and active transport of active ingredients, improve infiltration in narrow gaps, and hydration to the skin in lotions, sunscreens, deodorants, shampoos, conditioners, hair serums, and nail enamels [248]. 

### 10.3. Nanoparticles 

The nanoparticles differ in chemical compositions and morphologies. Nevertheless, they are used in sunscreen preparations (e.g., TiO_2_-nanoparticles, ZnO-nanoparticles, CeO_2_-nanoparticle, and ZrO_2_-nanoparticles) and physical UV filters [223]. In addition, silica and clay nanoparticles are added as thickeners [249,250].

The “gold nanoparticles” (range from 5 to 400 nm in size) display various forms (e.g., nanosphere, nanoshell, nanocube, nanostar, nanocluster, nanorod, and nano-triangles branched). They have essential characteristics such as non-cytotoxicity, inertness, highly stable nature, biocompatibility, antibacterial and antifungal properties. They are used in face packs, antiaging creams, deodorant, lotion, etc. [251].

The “lipid nanoparticles” (nanostructured lipid carriers (NLC) and solid lipid nanoparticles (SLN)) are used for the controlled release of actives and to improve skin hydration, enhancing the effect of occlusion. In addition, they improve the chemical stability of compounds light-sensitive and susceptible to hydrolysis and oxidation. Lipid nanoparticles are used to transport retinol, coenzyme Q10, tocopherol, and ascorbyl palmitate in cosmetics [252,253].

### 10.4. Silicone Matrices and Vesicles 

Silicones, in association with various active ingredients (e.g., aluminum, zirconium, and tetrachlorohydrex), can act as delivery vesicles for cosmetic actives. The silicon vesicles reduce stickiness and defend the actives from hydrolysis. Silicones are used in cosmetics for sunlight protection (stearyl dimethicone improves the sun-protection factor) and hair-care formulations since they enhance shine, conditioning, manageability, and decrease flyway [241].

### 10.5. Multi-Walled Delivery Systems

The multi-walled delivery system (MDS) mixes structured vesicle-forming ingredients and high-shear processing to give long-term stability to cosmetic formulations. Amphiphilic molecules (e.g., derivatives of polyglycerols, oleic acid, and amino acid residues) make MDS. As a result, MDS gives stability to liposomes and sustains and defends the skin, optimizing cosmetic product performance [254].

### 10.6. Emulsions

Some emulsion delivery systems (e.g., microemulsion, nanoemulsions, liquid crystal, multiple emulsions, and Pickering emulsions) are employed in cosmetics.

Microemulsions have a diameter < 100 nm. They are transparent (or translucent) dispersions of oil and water stabilized by surfactant/s molecules and co-surfactant/s. The surfactants have non-ionic groups, which determines their excellent cutaneous tolerance and balanced lipophilic and hydrophilic properties. The co-surfactants enhance interfacial fluidity, and regulate the Hydrophilic–Lipophilic Balance (HLB) of surfactants. Microemulsions are employed in the moisturizing formulation. They have an excellent aesthetic appearance, apply easily, and give no tackiness in the treated area [255]. Multifunctional silicone quaternary polymer microemulsions are used in hair-care formulation. They give protection from heat and conditioning, increase color retention, body volume, and product clarity [256]. Nanoemulsions have droplet diameter < 100 nm. They have good sensorial properties (e.g., merging textures, rapid penetration) and hydrating power. They are used in ringing gels, water-like fluids, transparent milk, lotions, and crystal-clear gels. Cationic nanoemulsions are employed in hair-care formulation to enhance the dry hair aspect (after several shampoos) [257].

Liquid crystals are a state of incomplete melting. They increase emulsion stability, act as rheological barriers to coalescence, and improve the cosmetic demand since the preparations into which they are incorporated have a colored appearance. Lipophilic materials into a liquid–crystalline matrix are protected from photo and thermal degradation [258]. Multiple emulsions are emulsions in which the dispersed phase encapsulate tiny droplets. The multiple emulsions can be Water/Oil/Water (*W*/*O*/*W*), in which external water phases are separated from an oil layer, and Oil/Water/Oil (*O*/*W*/*O*), in which water parts the two oil phases. In cosmetics, the most used type is *W*/*O*/*W.* They require two stabilizing surfactants, a low HLB (decaglycerol decaoleate, mixed triglycerol trioleate, or sorbitan trioleate forming a primary emulsion) and higher HLB surfactant (poloxamers and polysorbates to achieve the secondary emulsification) [259]. In cosmetics, they are used in personal care formulations containing skin lipids, perfumes, free radical scavengers, and vitamins [260]. Pickering emulsions are solid particles (e.g., zinc oxide or titanium dioxide) stabilized emulsions of water-in-oil (*w*/*o*), oil-in-water (*o*/*w*), or even multiple emulsions. They give a dry or dull impression on the skin, which the addition of cyclodextrin can overcome [261].

## 11. Conclusions

A significant correlation between the intake of food supplements and the skin’s wellbeing is reported in the literature. Unfortunately, currently, no specific legislation regulates their use as cosmetics. If many efforts have been made to improve the access of the active ingredients to the sites of use in our body through carriers that improve their bioavailability, there are no official or validated methods that allow us to identify and dose all the active ingredients obtained from food. A precise knowledge of this information would allow to maximize the cosmetic effects, reduce adverse reactions, and above all, it would help legislators formulate rules for the use of food-borne bioactive in cosmetic products.

## Figures and Tables

**Table 1:** Some food ingredients used in cosmetic formulations.

FOODS	Bioactive Molecules	Bioactivity	Cosmetic Relevance
*Green tea* 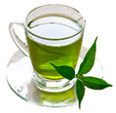	Catechin derivatives (e.g., epicatechin, epicatequinagalato, epigallocatechin, and epigallocatechin-3-gallate.	Free radical scavengers.	Green tea extracts have a prolonged moisturizing effect, improve microrelief, reduce skin roughness and sebum production, and prevent and treat acne vulgaris.
*Coffea arabica* 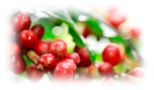	Proanthocyanidins, quinic acid, caffeic acid, and chlorogenic acid.	Antioxidant properties.	*Coffea arabica* extracts are skin-lightening agent and enhance wrinkle, fine line, and pigmentation in patients with actinic damage.
*Vitis vinifera* 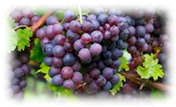	Stilbenes (e.g., resveratrol), proanthocyanidins, and procyanidins.	Antioxidant properties.	*Vitis vinifera* extracts inhibit UV light-mediated skin aging.
Pomegranate 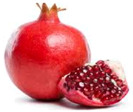	Ellagic acid, punicalagin, and punicic acid.	Antioxidant, antifungal, and anti-inflammatory properties.	Pomegranate extracts decrease wrinkles.
*Glycine max* (soybean) 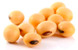	Isoflavones (e.g., genistein).	Antioxidant properties.	*Glycine max* extracts reduce UV-induced oxidative DNA damage and skin photodamage.
*Aloe vera* 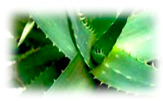	Aloesin, mucopolysaccharides, and amino acids (e.g., arginine, histidine, threonine, glycine, serine, and alanine).	Antioxidant, anti-inflammatory, and water-retention properties.	Soybean extracts have a skin-lightening effect, improve skin elasticity, and reduce wrinkles.
*Citrus limon* 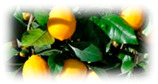	Flavanones (e.g., hesperidin), citral, D-limonene, and β-pinene.	Antioxidant properties.	*Citrus limon* extracts have antiaging and depigmenting effects, and reduce acne and hair disorders.
*Opuntia ficus indica.* 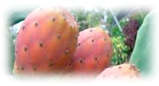	Linoleic acid, oleic, and stearic acid.	Stimulate cell renewal, supporting skin moisturizing and collagen production.	*Opuntia ficus indica* extracts have antiaging properties for skin, hair, and nails.
*Ficus carica.* 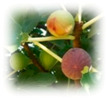	Ficin and phenolic compounds.	Antioxidant properties.	*Ficus carica* extract restores the regular epidermal, improves skin lightness, reduces sebum production, exfoliation, hyperpigmentation, wrinkle, acne, and freckles.
*Cynara scolymus.* 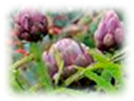	Phenolic compounds.	ROS-scavenging effect, anti-inflammatory effect, modulation of genes involved in antiaging processes.	*Cynara scolymus* extracts have a photoprotective effect and increase roughness and skin elasticity.
*Carica papaya.* 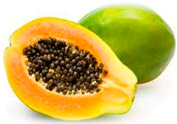	Flavonoids (e.g., kaempferol, quercetin, myricetin, and glycosides), phenolic acids (e.g., ferulic acid, caffeic acid), cysteine endopeptidases.	ROS=scavenging effect, and anti-inflammatory effects.	*Carica papaya* extracts reduce skin erythema, proteolytic wound debridement, and haveantibacterial effects.
*Glycyrrhiza glabra* (licorice). 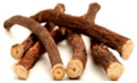	Flavonoids (e.g., glabridin, glabrene, isoliquiritigenin, licochalcone A, and liquiritin).	Antioxidant, anti-inflammatory, and modulation of diacetyl production.	*Glycyrrhiza glabra* extracts prevent pigmentation disorders (e.g., age spots, melasma, and sites of actinic damage) as deodorant properties and improve hair growth.
*Theobroma cacao.* 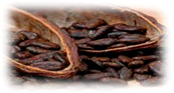	Polyphenols (e.g., flavan-3-ols, proanthocyanidins, anthocyanins) and methylxanthines (e.g., theobromine and caffeine).	Antioxidant and anti-inflammatory properties.	*Theobroma cacao* extracts have photoprotective properties and regulate collagen I, III, and IV and glycosaminoglycan production.
*Prunus dulcis* * 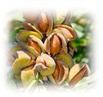 *	Triterpenoids (e.g., urosolic, betulinic, and oleanolic acids), catechin, flavonol glycosides, phenolic acids (e.g., protocatechuic acid and vanillic acid), phytosterol, fatty acids, and lipid-soluble vitamins.	Antioxidant properties.	*Prunus dulcis* extracts reduce eczema and pimples. Almond oil nourishes, softens, and strengthens the hair.
*Coconut* 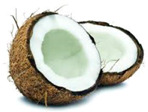	Fatty acids (e.g., myristic, lauric, and palmitic acids).	Antioxidant and anti-inflammatory properties.	Coconut oil inhibits UV light-mediated skin aging, moisturizes skin, reduces protein loss in the hair, is a useful scrub, and can be used as a deodorant. Coconut milk softens the skin and removes black spots.
